# Status and determinants of health behavior knowledge among the elderly in China: a community-based cross-sectional study

**DOI:** 10.1186/1471-2458-13-710

**Published:** 2013-08-02

**Authors:** Zhiqin Yin, Guiling Geng, Xuefen Lan, Liming Zhang, Surong Wang, Yuantong Zang, Meidi Peng

**Affiliations:** 1 Department of Nursing, School of Nursing, Wenzhou Medical College, Wenzhou 325035, China; 2 Department of Nursing, School of Nursing, Nantong University, Nantong 226019, China; 3 Wenzhou WuMa Community Health Service Center, Wenzhou 325000, China

**Keywords:** Community, The elderly, Health behavior, Knowledge, Children

## Abstract

**Background:**

Limited studies are available on health behavior knowledge among the elderly and the interaction between the elderly and their children living with them. Using a survey of the elderly in the community and their children living with them, we explored the characteristics that determine health behavior knowledge. We also identified the influencing factors affecting such knowledge. The relationship between the health behavior knowledge of the elderly and that of their children was also investigated.

**Methods:**

A total of 1207 elderly people were selected by multistage probability sampling (number of children of the elderly = 201). We used a self-designed behavior knowledge questionnaire (38 questions) to survey the participants. After the 38 questions were answered, the total score was determined. The group with adequate health behavior knowledge (score ≥ 24) and that with inadequate knowledge (score < 24) were distinguished by logistic regression to explain the influencing factors affecting health knowledge.

**Results:**

On the basis of the responses to 38 questions in the survey, approximately 46.7% of elderly people were identified as having a good knowledge of health. Knowledge of the proper amount of certain foods and liquids as well as that of psychological disorder among the elderly recorded a low percentage at < 60%. Factors related to health behavior knowledge among the elderly were as follows educational attainment, past occupation, and location of residence. Participants who finished high school or higher had 6, 4, and 3 times greater odds of possessing adequate health knowledge than those who attained below primary school, primary school, and junior high school levels, respectively. Those with experience as administrative and technical personnel, workers, migrant workers, and farmers had 2.5, 2.3, 3.9, and 2.1 times greater odds of possessing adequate health knowledge, respectively, than those who were unemployed. Respondents living in the city had 3.7 times greater odds of possessing adequate health knowledge than those living in the countryside. In the stem family, the health knowledge of the elderly was significantly lower than that of their children (P < 0.001). However, the influence of their children’s knowledge upper their elder’s was relatively weak.

**Conclusion:**

The need to improve health behavior knowledge among the Chinese elderly remains high. Medical personnel in the community should provide health education related to proper diet and alcohol intake, as well as psychological health, particularly for those elderly who only reached primary school and below, used to be unemployed, and are living in rural areas. Children of stem families should be guided to enhance their health education and contribute to the health knowledge of their elderly parents.

## Background

Health behavior and lifestyle are identified as the main factors affecting human health. Research shows that 45% of human diseases are related to personal behavior and lifestyle, and 60% of death is related to personal lifestyle [1]. Bad lifestyle and behavior are the pathogenic factors constituting 70% of the top 10 causes of diseases in the United States, whereas in China, the corresponding figure is 44.7% [2]. A report from the World Economic Forum (WEF) estimated that in the next 20 years, the economic loss caused by adverse behavior and lifestyle leading to chronic diseases would reach $4.7 billion [3]. Today, the number of patients with chronic diseases closely related to health behavior and lifestyle exceeds 260 million in China, and for the next 10 years, economic loss due to chronic diseases is predicted at 558 billion yuan [4].

Adverse health behavior and lifestyle affect all age groups (e.g., obesity in teenagers, heart disease in adults, cerebrovascular disease, malignant tumor, etc.), but its influence on the elderly is significant. Old people represent a population highly at risk for chronic diseases and fall injuries [5,6]. The elderly whose adverse health behavior and lifestyle led to diseases or fall injuries represented more than half of the population [5]. The consumption of health resources also had a considerable proportion. The third National Health Services investigation data in China indicated that the direct economic loss caused by cerebrovascular disease, malignant tumor, heart disease, diabetes, high blood pressure, and respiratory disease among people 65 years old or older reached 34 billion yuan [7]. The WHO reported that China had the largest incidence of fall-related injuries worldwide. The annual direct medical cost related to falls exceeds 5 billion yuan, with a social cost of up to 80 billion yuan [8]. Research shows that falls among the elderly are avoidable to a certain extent [9]. Some elderly people realize the risk of falls but have limited knowledge on how to prevent them, whereas others are not aware of the risk factors [10].

“Health” depends on an individuals’ understanding of the factors that affect it and our proper use of such “knowledge” in the prevention and treatment of a disease. The ability to use knowledge to promote health depends on our access to or awareness of reliable health information (Pakenham–Walsh, 2002) [11]. A recent survey revealed that health awareness among community Chinese elderly ranged from 40.14% to 73% [12-16]. In rural areas, most adults consist of old people (the mean age was 61.34 years with a standard deviation of 10), and approximately 25% of the people have ample health knowledge [12]. At present, the primary task of community health care workers remains focused on the improvement of health knowledge among the elderly.

To improve the health behavior of the elderly, enhancing their health knowledge, increasing their confidence in maintaining their health, and strengthening their social environment are important [17]. Traditional family patterns or trunk families (a family in which the parents and a pair of married children live together [18]) are still common in China, given its strong family ties. Many intervention studies of elderly family members have shown that [19,20] the promotion of health knowledge among family members positively influenced health knowledge and disease rehabilitation among the elderly. Health education, increased health knowledge, and promotion of the positive effects of a healthy lifestyle were key to improving the health behavior of the Chinese elderly.

The Yangtze River Delta in China has the largest fastest-aging population in the country. The aging population has reached approximately one-fifth of the total population in this region, whereas the elderly population of Zhejiang and Jiangsu provinces have exceeded the provinces’ total population by 17% [21,22]. The question lies in how to improve the health knowledge and health behavior of this large elderly group, as well as prevent and delay diseases and disabilities related to health behavior and lifestyle. Current health behaviors and deficiencies in health knowledge should be identified. The influence of family members on one another in terms of health knowledge should also be determined.

Most community studies on the health knowledge of the elderly have thus far focused on the characteristics of old people. The mutual influence between family members, particularly between the elderly and their children who live with them, have not been considered. In addition, some survey questions regarding the health knowledge of the elderly involved only certain aspects of health behavior [12-16]. Some studies have verified that age, sex, educational level, previous employment, location of residence, and history of chronic diseases significantly influenced the health knowledge of the elderly [12-16,23]. Overall, intervention studies on teenagers living in UK and families of the elderly’s children have confirmed that health education for the main members of the family positively influenced the health behavior of the target group, that is, the patients [24]. This result is attributed to the tendency among family members to share similar lifestyles and habits [1]. Consequently, we strengthened our definition of health behavior, comprehensively evaluated the level and determinants of health behavior knowledge among the elderly, and analyzed the key factors influencing the health behavior knowledge of the children living with their elderly parents. The research questions of the current study are as follows: (1) What is the level of health behavior knowledge of the elderly people in the Yangtze River Delta (e.g., Wenzhou and Nantong) community? (2) What factors are associated with the health behavior knowledge of the community elderly? (3) Do elderly people have higher level of health behavior knowledge than their children living with them? What is the degree of mutual influence between the two groups?

## Methods

### Study objects

Participants included senior citizens aged 60 and above living in Wenzhou City in Zhejiang Province and Nantong City in Jiangsu Province. In accordance with the principle of sample size estimation, the sample size obtained was slightly greater than that obtained by simple random sampling (formula: n = Uα^2^ PQ/δ^2^) [17]. α was set at 0.05; p = 60%. According to the goal of the National Health Promotion Project for Hundreds of Millions of Chinese Farmers (NAHPF), δ = 0.05 P. Response rate was set at 85%. The sample size was determined to be 1024. The representative sample was obtained by multistage probability sampling. In each urban district, poor and good economic areas were selected based on Wenzhou and Nantong statistics from 2010. We selected a street in each area by simple random sampling. Using the same method, we chose a street from an urban and rural community. Each community was selected using this technique to select a neighborhood or a village. Four neighborhood committees and four villages were finally selected. Elderly people residing in these areas were investigated.

As urban centers with fast economic development, Wenzhou City and Nantong City have seen a rapidly growing immigrant population in recent years. In Wenzhou, the immigrant population comprises more than 30% of the city population. The corresponding percentage in Nantong is 15%. The immigrant population primarily originates from the following regions of China: Southwest China, Northwest China, Northeast China, and Central China [25,26]. In the present study, the samples include the original local elderly and the immigrant elderly, which can represent to a certain extent the health behavior knowledge of most elderly people in China.

### Study design

A community-based cross-sectional study was conducted from November 2011 to March 2012. We used self-designed questionnaires (see Additional file 1: Table S1) to investigate these randomly selected elderly people. The questionnaire solicited social demographic data and health behavior knowledge. This health behavior knowledge was formulated by the definition of health behavior with reference to the basic health knowledge and skills of Chinese citizens published by China’s Ministry of Health and “NAHPF”. The questionnaire included questions regarding diet, exercise, sleep, personal hygiene, hobbies, fall prevention, and psychological health affecting the elderly. The aspects of the problem were classified into seven and a total of 38 items.

The questionnaire was examined by nine experts and was pre-tested among 95 elderly people prior to modification. The content validity index of the questionnaire was calculated at 0.979, and the internal consistency measured with the Kuder–Richardson Formula 20 was 0.845. The main interviewers consisted of the task group members and 40 nursing students who underwent professional training before the survey and could speak the dialect.

Our study combined centralized surveys with in-home interviews. We interviewed every elderly person to obtain data after the elderly had undergone physical examination, which helped improve the efficiency of our investigations. Our investigations and physical tests were conducted by medical personnel, community health workers, and junior students.

The elderly people and their children were surveyed to determine the difference in health behavior knowledge between the former and the latter. The questionnaires were taken home by the elderly and filled out by their children. After two weeks, the community staff went to the homes of the surveyed elderly individuals to collect the questionnaires. The questionnaires that were distributed to the elderly and their children contained the same questions assessing the status of health behavior knowledge of the elderly; however, the instructions and the section for social demographic data were modified.

This study was conducted in compliance with the Helsinki Declaration and reviewed and approved by the Wenzhou medical college ethics committee. The community leaders signed a letter of consent, and verbal consent (as many of the old people could not write) was obtained from the elderly. The respondents were assured that all data regarding the elderly would remain confidential and only be used for the purpose of the research.

### Operational definition

Elderly: people aged 60 and above in developing countries and the Asia-Pacific region, as defined by WHO [27]. Health behavior in this study is defined as people’s engagement in activities to improve their health, maintain and promote physical and mental health, and avoid diseases [28], including daily health habits (adequate nutrition, sufficient sleep, proper exercise, personal hygiene, etc.), health behavior (regular health checks, actively seeking medical advice, etc.), preventive behavior (avoiding environment and events with negative health effects), change in health behavior (to discontinue smoking, drinking, etc.). Knowledge is defined as the identification of all entities and properties [29].

### Data analysis

All data were entered in duplicate into the EpiData version 3.1 database and statistics program (Atlanta, Georgia, USA), and data entry screens were used to revise incorrect entries (i.e., logic and input errors). Statistical analysis was performed using SPSS 14.0 (SPSS, Chicago, IL, USA). The overall score for health behavior knowledge was determined based on the sum of the correct responses to the 38 questions related to health behavior. Each correct answer earned one point. Incorrect, missing, or “don’t know” answers were awarded no points. The total number of correct answers ranged from 0 to 38 points. The NAHPF [30] reported on the low index of health behavior knowledge among the community elderly. In the present study, the cutoff score distinguishing adequate versus inadequate health behavior knowledge was set at 24 (more than 60% correct answers). Respondents with a cutoff score (i.e., 24 or more items answered correctly) or above were considered to have adequate health behavior knowledge, whereas those who earned a score below the cutoff score (i.e., 23 or fewer correct responses) were considered to have inadequate health behavior knowledge. The independent variables of this study included individual-level factors (sex, age, culture level, and previous career), family factors (people members they live with), disease factors (whether suffering from chronic diseases or not) and regional factors (residence), which were summarized using descriptive statistics. We used the chi-squared test to analyze these variables to determine the difference between the group with adequate health behavior knowledge and that with insufficient knowledge. The chi-squared test was also used to analyze the difference in health behavior knowledge between the elderly and their children.

Logistic regression models were used to control for potential confounding factors. In all regression models, the outcome variable was the adequacy or level of health behavior knowledge (if 24 or over, then y = 1; otherwise, y = 0). The independent variables in the single-factor analysis consisted of meaningful variables. The significance level (a) was set at 0.05.

## Results

### Demographic characteristics of the elderly

A total of 1,281 questionnaires were distributed, of which 1,271 were returned. Among the returned questionnaires, 1,207 were considered valid responses (The effective rate was 94.2%). A total of 64 elderly people were excluded because of invalid or incomplete responses replies. Among the respondents, 529 (43.8%) were male, and 678 (56.2%) were female. The mean age was 71.42, and the standard deviation was 7.29. A total of 476 (39.4%) respondents lived in urban areas, whereas 731 (60.6%) lived in the rural areas. Other social demographic data are presented in Table 1.

**Table 1:** Social demographic data (Number of respondents = 1207)

**Characteristics**	**N (%)**	**Characteristics**	**N (%)**
**Age**		**Education level**	
60 to 69	524 (43.4)	Primary school or below	506 (41.9)
70 to 79	504 (41.8)	Primary school	327 (27.1)
≥80	179 (14.8)	Middle school	194 (16.1)
**Living with whom**		High school and above	180 (14.9)
Self	162 (13.4)	**Previous occupation**	
Spouse	753 (62.4)	Administrative/Technical	270 (22.4)
Children and family	270 (22.4)	staff	
Other people	22 (1.8)	Factory workers	299 (24.8)
**Marital status**		Migrant workers	45 (3.7)
Married	918 (76.1)	Farmers	436 (36.1)
Unmarried/Divorced	11 (0.9)	No work	157 (13)
Widowed	278 (23.0)	**Chronic diseases**	
		No	456 (37.8)
		Yes	751 (62.2)

### Status of health behavior knowledge among the elderly

Among the 1,207 old people who were interviewed, the mean and the standard deviation of the number of correct answers were 22.96 and 5.08, respectively. Given a cutoff of 24, of the 575 participants, 47.6% responded correctly. This percentage represented the elderly with adequate health behavior knowledge. The number of respondents with inadequate health behavior knowledge was 632 (52.4%). The mean of the items correctly answered by the group with adequate health behavior knowledge was 27.18 (SD 3.48), and that of the inadequate knowledge group was 19.11 (SD 2.59).

**Figure 1:**
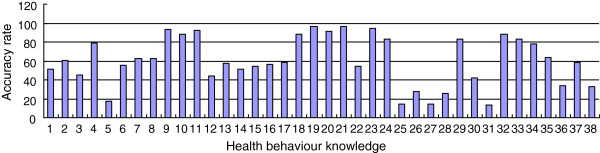
**Accuracy rate of the responses to each health behavior knowledge item among the elderly.** Note: 1 Exercise. 2 Frequency of exercise. 3 Duration of exercise. 4 Intensity of exercise. 5 Exercise schedule. 6 Amount of sleep. 7 Sleep quality. 8 Side effect of sleeping pills. 9 Slippery floors. 10 Indoor lighting. 11 Toilet safety. 12 Locking the bathroom while bathing. 13 Height of bed. 14 Uphill fetch. 15 Anti-skid motion. 16 Speed of movement. 17 Speed of rising. 18 Washing hands before meals. 19 Washing hands after using the toilet. 20 Cutting nails frequently. 21 Use of own towel. 22 Frequency of brushing the teeth. 23 Ventilation. 24 Hazard of smoking. 25 Liquor intake. 26 Beer intake. 27 Cooking oil intake. 28 Salt intake. 29 Meat intake. 30 Vegetable intake. 31 Fruit intake. 32 Diet regularity. 33 Diet intake. 34 Interaction/Communication. 35 Solitary lifestyle. 36 Diagnosis of dementia. 37 Treatment of dementia. 38 Diagnosis of old-age depression.

The overall status of health behavior knowledge (shown in Figure 1 from left to right) showed that knowledge related to exercise (items 1 to 5) was low, except for “physical activity”. For the other entries, the accuracy rate ranged from 17.3% to 55.8%; among these, the accuracy rate of the responses to the item regarding period of exercise earned the lowest rate. The accuracy rate of responses regarding sleep as a factor (items 6 to 8) was 60%. In terms of fall prevention (items 9 to 17), the elderly exhibited a satisfactory understanding of the environmental factors leading to fall-related injuries (accuracy rate was 87.8% to 93.5%) but a low level of knowledge of the behavior factors that lead to fall injuries (accuracy rate was 60%). Except for frequency of brushing, daily health habits (items 18 to 23) earned the highest accuracy rate. The rest of the items achieved accuracy rates ranging from 88.1% to 95.9%. The responses to questions related to the hazard of smoking (item 24) obtained an accuracy rate of 82.9%. Mastery of diet knowledge (items 25 to 33) was found to exhibit the poorest accuracy rate. This item included knowledge regarding proper/maximum intake of certain foods and drink (e.g., intake of oil, salt, fruits, vegetables, and liquor) obtained the lowest accuracy rate from 13.4% to 41.6%. The elderly demonstrated satisfactory understanding of the effects of social interaction (items 34 to 35) as well as solitary life (items 36 to 38), with accuracy rates of 78.2% and 64%, respectively. However, the elderly showed insufficient understanding of dementia and depression, obtaining an accuracy rate ranging from 32.7% to 58.2%.

### Factors affecting health behavior knowledge among the elderly

We used single-factor analysis to analyze the social demographic factors affecting the health behavior knowledge of the elderly. Except gender and chronic diseases, age, marital status, educational level, previous occupation,living companions and residential factors were closely related to health behavior knowledge among the elderly (P value < 0.05), as shown in Table 2.

**Table 2:** Single-factor analysis of items related to good health behavior knowledge among the elderly

**Predictor**	**Group with adequate health knowledge**		**Group with inadequate health knowledge**		***X***^**2**^	**P**
	**N**	**%**	**N**	**%**		
**Sex**					0.013	0.908
Male	253	47.8	276	52.2		
Female	322	47.5	356	52.5		
**Age**					8.434	0.015
60 to 69	272	51.9	252	48.1		
70 to 79	231	45.8	272	54.2		
≥80	72	40.2	107	59.8		
**Marital Status**						
Married	461	50.2	457	49.8	10.389	0.006
Widowed	109	39.2	169	60.8		
Divorced/Unmarried	5	45.5	6	54.5		
**Education level**						
Primary school or below	153	30.2	353	69.8	199.691	0.000
Primary school	141	43.1	186	56.9		
Middle school	123	64.4	71	36.6		
High school and above	158	87.8	22	12.2		
**Previous occupation**						
Administrative/Technical staff						
Factory workers	203	75.2	67	24.8	198.022	0.000
Migrant workers	183	61.2	116	38.8		
Farmer	20	44.4	25	55.6		
	138	31.7	298	68.3		
No work	31	19.7	126	80.3		
**Living with whom**						
Self	59	36.4	103	63.6	9.663	0.022
Spouse	375	49.8	378	50.2		
Children and family	130	48.1	140	51.9		
Other people	11	50.0	11	50.0		
**Chronic diseases**						
No	212	46.5	244	53.5	0.387	0.553
Yes	363	48.3	388	51.7		
**Residence**						
City	352	73.9	124	26.1	236.076	0.000
Rural	223	30.5	508	69.5		

To control the known confounding factors, we entered the significant variables determined by single-factor analysis into the regression equation and conducted the multivariate logistic regression analysis. Results indicated that the factors below were significantly associated with elderly health behavior knowledge. As shown in Table 3, respondents who attained high school education or above showed good health behavior knowledge 6, 4, and 3 times higher compared with those who attended up to primary school or below (OR = 0.166, 95%; CI = 0.09, 0.29), primary school (OR = 0.247, 95% CI = 0.14 0.44), and junior high school (OR = 0.337, 95% CI = 0.19–0.60), respectively. Regarding previous occupation, administrative and technical personnel, professional workers, migrant workers, and farmers exhibited good health behavior knowledge higher than those of respondents with no occupation by 2.5 (OR = 2.466, 95% CI = 1.38 to 4.41), 2.3 (OR = 2.292, 95% CI = 1.36 to 3.86), 3.9 (OR = 3.852, 95% CI = 1.87 to 7.95), and 2.1 times (OR = 2.119, 95% CI = 1.33 to 3.37). Meanwhile, compared with those who lived in rural areas, respondents in urban areas were 3.5 times more likely (OR = 3.524, 95% CI = 2.36 to 5.20) to possess adequate health behavior knowledge.

**Table 3:** Logistic regression analysis of relevant factors affecting knowledge of good health behavior among the elderly

**Variables**	**Reference value**	**B value**	**SE**	**Wald**	**P value**	**OR**	**95% CI**	
							**Lower limit**	**Upper limit**
**Education level**	High							
Primary school or below	school and above							
		−1.793	0.290	36.19	0.000	0.166	0.09	0.29
Primary school		−1.399	0.293	22.80	0.000	0.247	0.14	0.44
Middle school		−1.081	0.291	13.97	0.000	0.337	0.19	0.60
**Previous occupation**								
Administrative/	No work	0.903	0.297	9.24	0.002	2.466	1.38	4.41
Technical staff								
Factory workers		0.829	0.266	9.74	0.002	2.292	1.36	3.86
Migrant workers		1.349	0.370	13.30	0.000	3.852	1.87	7.95
Farmers		0.751	0.237	10.07	0.002	2.119	1.33	3.37
**Residence**								
Rural	Urban	−1.260	0.198	40.42	0.000	3.524	2.39	5.20

### Comparison of health behavioral knowledge between the elderly and their children

A total of 270 questionnaires on health behavior knowledge were distributed to children living with their elderly parents. Of this total, 201 valid questionnaires were received. (The effective rate was 74.4%, because 12 questionnaires showed invalid answers, and 57 had no answers). Among the respondents, 136 (67.7%) were male and 65 (32.3%) were female. The mean age was 43.90, and the standard deviation was 8.70. Of the total, 68 and 133 elderly people lived in urban and rural areas, respectively. Furthermore, 43 respondents obtained primary school education, 103 attended junior high school, 45 finished high school and above. A total of 30 respondents were below 35 years old, 115 were between 35 and 50, and 56 were older than 50 years.

The mean and the standard deviation of the correct responses to health behavior knowledge of 201 children were 29.4 and 3.17, respectively. Using the same cutoff score (24) as that of the elderly, we distinguished the group with sufficient behavior knowledge from that with insufficient behavior knowledge. A total of 193 participants (96%) exhibited adequate health behavior knowledge, and 8 (4%) had insufficient knowledge. The mean number of correct responses of the group with adequate knowledge was 29.75 (SD 2.70), and that with inadequate knowledge was 21.0 (SD 1.93). Among the 201 elderly people, 82 (40.8%) showed good knowledge, and 119 (59.2%) showed insufficient knowledge.

**Figure 2:**
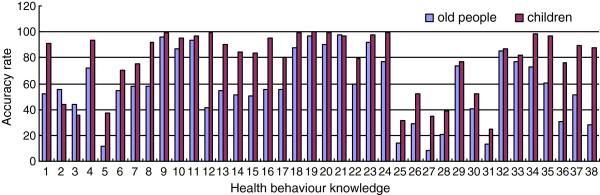
**Accuracy rate of responses to each health behavioral item among the elderly and their children.** Note: This diagram is as shown in Figure 1.

The health behavior knowledge of the elderly was markedly lower than that of their children (*X*2 = 141.82, P < 0.001). Figure 2 presents the responses of the elderly and their children to the health behavior knowledge items. Items regarding factors that lead to falls (items 9 to 11), daily health habits (items 18 to 21 and 23), diet regularity (item 32) obtained a good accuracy rate (> 80%), food and liquor intake (e.g., oil, salt, vegetables, fruits, alcohol) (items 25 to 28), and the proper exercise time (5 items) obtained a low accuracy rate (< 40%). The elderly’s children also showed better knowledge in the mental health aspect compared with their elderly parents(accuracy rate 76.1%-89.1%). These results and Figure 1 can be used as references by community health departments in formulating health measures for the elderly and their family members.

## Discussion

In this study, more than 50% of the participants exhibited inadequate health behavior knowledge, including all elderly persons. These finding was consistent with those of other studies [30-33] but higher than the findings in the study by Tian et al. [13,34] regarding the health knowledge of rural elderly people about chronic diseases.

In general, more than 60% of the elderly showed adequate knowledge of the factors that induce falls, as well as the importance of daily health habits, regular diet, and exercise. In addition, the respondents understood the danger of smoking, importance of social interaction, and effects of living alone. Knowledge of the proper/maximum intake of oil, salt, fruits, vegetables, and liquids, as well as information on elderly depression, need to be strengthened (with less than 60%). Since 1953, the government of the People's Republic of China has implemented disease prevention programs as the focus of health knowledge promotion. In the past 20 years, the government and the Department of Health Education have provided sufficient attention to health education as well as healthy behavior and lifestyle. Thus, most people have acquired adequate knowledge of the components that complete a healthy lifestyle [2]. Inadequate orientation to healthy habits and lifestyle lead to poor understanding of the importance of using appropriate amounts of cooking oil and salt in preparing food, realizing the benefits of appropriate intakes of vegetable, fruits, and liquids, as well as recognizing symptoms of common mental disorders. Thus, these aspects of health knowledge should be the focus of future efforts in health education.

Studies worldwide found that people aged 65 years or older with lower educational level had less health behavior knowledge [35,36]. A close relationship was found between the elderly health knowledge and their educational level; the higher the educational level, the better their health knowledge [37,38]. The present study confirmed that the participants who attained high school level or above had 6, 4, and 3 times greater odds of possessing adequate health knowledge than those who attained below primary, primary, and junior high school education, respectively. High reading comprehension level, broad knowledge, analytical ability, and screening information ability were better in people who obtained higher education compared with lower education, which may explain more accurately the similar results [39].

The current study also revealed that the elderly without previous employment exhibited significantly lower health knowledge than those who were previously employed (participants who used to work as administrative and technical personnel, workers, migrant workers, and farmers had 2.5, 2.3, 3.9, and 2.1 times greater odds of possessing adequate health knowledge than those who were unemployed, respectively). This finding was consistent with the results of other previous studies [16]. People with different occupations had various social interaction as well as access to and distribution of information. Among the Chinese elderly, those who worked as administrative and technical workers obtained a higher level of health behavior knowledge because they had more access to information and knowledge was more easily disseminated in their profession [40]. Those elderly who did not previously hold permanent occupations showed a low educational level, and primarily did the household chores; these people had limited access to information and had fewer ways of acquiring knowledge. In China, more than 40% of the elderly received no basic education, particularly females [41]. The present study confirmed this percentage. Because most respondents were born before 1950, and poor standard of living rendered them incapable of pursuing formal education.

A significant difference in health literacy has been indicated between the Chinese elderly living in urban areas and those living in rural areas [42]: the elderly living in urban areas showed significantly higher health behavior knowledge than did the rural elderly; the number of elderly persons living in cities and exhibiting good health behavior knowledge was 3.5 times greater than those living in rural areas. This result may be attributed to differences in economic conditions, educational attainment, and health resources between those living in urban and rural areas. According to the Rural Health Resource Allocation Report in 2010 [43], the current health resources of China are mainly allocated to hospitals at or above the county level. Township institutes are generally less prestigious and less sophisticated in terms of technological facility. In addition, their technical personnel generally obtained relatively low educational degrees. Excellent community medical care, health care services, and health education are difficult to provide because of shortage in technical health personnel in town hospitals. Therefore, prompted community health departments should increase the number of skilled personnel in towns and restructure the health department to strengthen health education among rural people, particularly the elderly.

In previous studies, gender and marital status were factors that affecting health knowledge of the elderly. Male, married people have better health knowledge than the one who was female, divorced or widowed [13,15,16], but this study do not show this difference, which must be confirmed in future research.

A study showed [16] that the level of health knowledge among the elderly who were chronically ill was higher than those without chronic diseases. However, these results were contrary to the findings in the present study, which reveals no difference in health behavior knowledge between the elderly who were chronically ill and those who were not. We found that this variation could be attributed to the content of the survey items. The previous study investigated the general health and chronic disease-related knowledge among the elderly, whereas the present study focused on general health knowledge (daily health behaviors). This study also found that those elderly with chronic diseases were more likely to acquire knowledge related to chronic diseases.

With the rapid urbanization in China and the migration of the rural population to the city in recent years, the number of the elderly who left the rural areas increased [44], and the number of rural stem families declined. This study indicated that the number of old people living with their children was less than one-fourth of the investigated number.

In the present study, the comparison of the health behavior knowledge between the elderly and their children indicated that both groups exhibited a tendency to improve their health behavior knowledge. For example, the elderly possessed adequate health knowledge of the factors that induce falls, as well as the importance of daily health habits, regular diet, and daily exercise. The elderly were also aware of the dangers of smoking, importance of social interaction, and effects of living alone (with an accuracy rate above 80%). However, their knowledge regarding the following must also be strengthened: appropriate amounts of oil and salt used in food preparation; intake of fruits, vegetables, and liquids; and symptoms of early depression (with an accuracy rate below 40%). Family interaction regarding their collective health knowledge also contributed to the improvement of health behavior knowledge. Living in the same environment over a long period of time allowed the members to subtly influence one another.

In addition, the health behavior knowledge of the elderly was significantly lower than that of their children (*χ*^2^ = 141.82, P < 0.001). Knowledge of mental health prominently showed that family members influence one another in terms of health knowledge. Despite the seemingly dominant roles of the elderly in the family, they may not be sufficiently strong to promote solid health knowledge in the family. Logistic regression analysis of the influencing factors of health knowledge among the elderly also confirmed this view. (The item stating “with whom to live together” Was removed from the questionnaire). We interviewed some of the elderly and their children, and the general view of the elderly was that the health behavior knowledge was similar to knowledge of daily life such that they perceived information coming from medical staff to be more credible than information coming from their children. The reason for this observation may be that the elderly had more trust in the medical staff’s experience. The children’s common view was that they had significant health behavior knowledge but that they did not pay sufficient attention to them because the habits formed by long-term living with their parents would weaken their trust in this knowledge.

Accordingly, community health workers should emphasize the importance of education and health awareness and make the children become actively involved and responsible in the implementation of overall family education.

## Conclusions

This study confirms the need to fill the gap in health behavior knowledge among the Chinese elderly, given that only 47.6% of the elderly possessed adequate health knowledge. Medical personnel in the community should provide health education regarding diet and alcohol, as well as information regarding psychological health, particularly targeting those elderly who were only able to reach primary school and below, used to be unemployed, and living in rural areas. Guidance should likewise be provided to children of stem families to expand their health education and promote health behavior knowledge among their elderly parents.

### Limitation

The study sample included elderly people residing in Wenzhou City and Nantong City in China. The study samples were obtained from these cities. Migrant population comprised more than 30% in Wenzhou and about 15% in Nantong. The study results represented only a portion of the elderly. Elderly people from other regions may not have been properly represented to a certain extent because of the variations in regional economic and cultural development in China. The low accuracy rate of the elderly living with their children may have affected the difference in the health behavior knowledge between the elderly and their children. This limitation has to be resolved by improving and confirming the response rate of the children of the elderly in future research. Although income may also affect health behavior knowledge, most elderly people could not accurately answer this question. Thus, this variable was not included in the study, which may have affected the results.

## Competing interests

The authors declare that they have no competing interests.

## Authors’ contributions

ZQY designed the study, conducted the data analysis, and completed the first draft of this article. GLG participated in the design of the study and coordination, data collection. XFL participated in data collection, and paper editing and revising. LMZ, SRW, YTZ, MDP participated in data collection, All authors have read and approved the final manuscript.

## Pre-publication history

The pre-publication history for this paper can be accessed here:

http://www.biomedcentral.com/1471-2458/13/710/prepub

## Supplementary Material

Additional file 1: Table S1 Health behavior knowledge questionnaire of the elderly. Click here for file
