# Supplementary material for: Status and determinants of health behavior knowledge among the elderly in China: a community-based cross-sectional study
Source: BMC Public Health. 2013 Aug 2;13:710. doi: 10.1186/1471-2458-13-710 (PMC3751703; doi:10.1186/1471-2458-13-710)
Supplement: Additional file 1: Table S1 — Health behavior knowledge questionnaire of the elderly. [file 1471-2458-13-710-S1.doc]

# Additional files

### Health behavior knowledge questionnaire of the elderly

**Social demographic data**

| 1. Gender：①Male ②Female |
| --- |
| 2. How old are you? |
| 3. Who are you living with? ①Nobody ②Spouse ③Children ④Others |
| 4. Marital Status: ①Single ②Married ③Divorce ④Widowed |
| 5. Educational level:①Less than basic education ②Basic education ③Junior school  ④High school and above |
| 6. What was your previous occupation?: ①Administrative and technical staff ②Workers ③Migrant workers ④Farmers ⑤Unemployed |
| 7. Where do you live？①Urban ②Rural |
| 8. Do you have a chronic disease? ①No ②Yes (Specify the illness .) |

Knowledge of elderly health behavior questionnaire

| 1. Can the elderly do strenuous exercise? ①Yes ②No ③Don’t know |
| --- |
| 2. How many times per week should the elderly exercise? ①1-2 ②≥3 ③Don’t know |
| 3. How long should the elderly exercise each time? ①≤ 30 min ②≥ 30 min ③Don’t know |
| What indicates an appropriate extent of exercise? ①Perspiring or mild fatigue ②Moderate fatigue ③Don't know |
| Which is the best time to exercise? ①Morning ②Forenoon ③Afternoon ④Evening ⑤Don’t know |
| How long is the sleep time of the elderly? ①4 h to 5 h ②6 h to 8 h ③ > 8 h ④Don’t know |
| 7.Which situation provides quality sleep? ①Sleep for a long time ②Sleep for a short time ③Relax after sleep ④Don’t know |
| 8. Can hypnosis affect human health? ①Yes ②No ③Don’t know |
| 9. Does slippery floor cause falls in the elderly? ①Yes ②No ③Don’t know |
| 10. What amount of light in the elderly’s bedroom is appropriate? ①Bright ②Moderate ③ Very bright ④Don’t know |
| 11. Between a pedestal toilet and a squat toilet, which is safer? ①Pedestal toilet ②Squat toilet ③Don’t know |
| 12. Must the elderly lock the door when bathing? ①Yes ②No ③Don’t know |
| 13. Dose the height of the bed cause falls among the elderly? ①Yes ②No ③Don’t know |
| 14. Can the elderly ascend? ①Yes ②No ③Don’t know |
| 15. Should the elderly wear boots when they exercise? ①Yes ②No ③Not matter ④Don’t know |
| 16. Does working quickly cause falls among the elderly? ①Yes ②No ③Don’t know |
| 17. What should the elderly do after wake up? ①Get up at once ②Get up after a few minutes ③Don’t know |
| 18. Must the elderly wash hands before meals? ①Yes ②No ③Don’t know |
| 19. Must the elderly wash hands after going to toilet? ①Yes ②No ③Don’t know |
| 20. Must the elderly trim the nails frequently? ①Yes ②No ③Don’t know |
| 21. Must family members use their own towel? ①Yes ②No ③Don’t know |
| 22. How many times a day should you brush your teeth? |
| 23. Must windows be opened frequently? ①Yes ②No ③Don’t know |
| 24. Is smoking harmful to the body? ①Yes ②No ③Don’t know |
| 25. How much (ml) liquor could we drink everyday? |
| 26. How much (ml) beer could we drink everyday? |
| 27. How much (ml) edible oil could we consume everyday? |
| 28. How much (g) salt could we consume everyday? |
| 29. Would eating more meat benefit humans? ①Yes ②No ③Don’t know |
| 30. How much (g) vegetable should we eat everyday? |
| 31. How much (g) fruits should we eat everyday? |
| 32. Should we have meals at regular hours and in a fixed quantity? ①Yes ②No ③Don’t know |
| 33. What duration of eating is appropriate for the elderly? |
| 34. Should the elderly communicate with people? ①Yes ②No ③Don’t know |
| 35. Should the elderly be often left alone at home? ①Yes ②No ③Don’t know |
| What is the main symptom of early dementia? ①Easily forgetting recent events ②Easily forgetting past events ③Don’t know |
| 37. Does dementia need treatment? ①Yes ②No ③Don’t know |
| 38. What is the symptom of elderly depression (Multi-choice)? ①Gloomy mood ②Do not want to speak ③Do not want to do anything ④Don’t know |
